# Euro-Collins Solution Versus Uw-Solution for Long-Term Liver Preservation in the Isolated Rat-Liver Perfusion Model

**DOI:** 10.1155/1991/10965

**Published:** 1991

**Authors:** René Den Toom, Marion De Jong, Eric P. Krenning, Hans J. Van Der Hoek, Fiebo J. W. Ten Kate, Georg Hennemann, Onno T. Terpstra

**Affiliations:** 1 Departments of Surgery University Hospital“Dijkzigt” Erasmus University Rotterdam Dr Molewaterplein 40 Rotterdam 3015 GD The Netherlands; 2 Departments of Internal Medicine III University Hospital“Dijkzigt” Erasmus University Rotterdam Rotterdam The Netherlands; 3 Departments of Pathology University Hospital“Dijkzigt” Erasmus University Rotterdam Rotterdam The Netherlands

## Abstract

To compare UW-solution (UW) and Euro-Collins (EC) for long-term liver preservation we investigated the morphology and metabolic capacity of rat liver after 18 and 42-hours cold-storage in either UW or EC.

After harvesting the rat liver was transferred to a perfusion chamber where it was perfused for 10 min with UW or EC at 4°C. Thereafter livers were stored at 4°C in UW or EC for 18 hours (both groups n = 6) or for 42 hours (both groups n = 8). After 18-hr or 42-hr cold-storage a 2-hr warm perfusion (37°C) was started with Krebs-Ringer solution with carbogen to which ^125^Iodine-triiodothyronine (T_3_) was added. Control livers (n = 8) were immediately perfused with Krebs-Ringer without cold-storage. The following parameters were assessed: ASAT-levels in the perfusate, T_3_-metabolites in the bile and the perfusate, the perfusion pressure, the volume of bile secreted and light-microscopical morphology at the end of the warm perfusion period.

After cold storage in UW-solution the ASAT-levels in the perfusate were lower than after storage in EC as well as the perfusion pressures. These livers demonstrated a better T_3_-metabolism and secreted more bile than EC-stored livers. Histological examination showed more tissue damage in the EC-stored livers than in the UW stored livers.

We conclude that cold-storage of rat liver in UW-solution resulted in a better morphology and metabolic capacity as compared with EC-solution.


*HPB Surgery*, 1991, Vol. 4, pp 313–320
Reprints available directly from the publisher
Photocopying permitted by license only

© 1991 Harwood Academic Publishers GmbH
Printed in the United Kingdom

### EURO-COLLINS SOLUTION VERSUS UW-SOLUTION FOR LONG-TERM LIVER PRESERVATION IN THE ISOLATED RAT-LIVER PERFUSION MODEL

RENÉ DEN TOOM[1], MARION DE JONG[2], ERIC P. KRENNING[2], HANS J. VAN DER HOEK[2], FIEBO J.W. TEN KATE[3], GEORG HENNEMANN[2] and ONNO T. TERPSTRA[1]

*Departments of Surgery[1], Internal Medicine III[2] and Pathology[3], University Hospital "Dijkzigt", Erasmus University Rotterdam, Rotterdam, The Netherlands*

*(Received 15 March 1991)*

To compare UW-solution (UW) and Euro-Collins (EC) for long-term liver preservation we investigated the morphology and metabolic capacity of rat liver after 18 and 42-hours cold-storage in either UW or EC.

After harvesting the rat liver was transferred to a perfusion chamber where it was perfused for 10 min with UW or EC at 4°C. Thereafter livers were stored at 4°C in UW or EC for 18 hours (both groups n = 6) or for 42 hours (both groups n = 8). After 18-hr or 42-hr cold-storage a 2-hr warm perfusion (37°C) was started with Krebs-Ringer solution with carbogen to which $^{125}$Iodine-triiodothyronine ($T_3$) was added. Control livers (n = 8) were immediately perfused with Krebs-Ringer without cold-storage. The following parameters were assessed: ASAT-levels in the perfusate, $T_3$-metabolites in the bile and the perfusate, the perfusion pressure, the volume of bile secreted and light-microscopical morphology at the end of the warm perfusion period.

After cold storage in UW-solution the ASAT-levels in the perfusate were lower than after storage in EC as well as the perfusion pressures. These livers demonstrated a better $T_3$-metabolism and secreted more bile than EC-stored livers. Histological examination showed more tissue damage in the EC-stored livers than in the UW stored livers.

We conclude that cold-storage of rat liver in UW-solution resulted in a better morphology and metabolic capacity as compared with EC-solution.

KEY-WORDS: Liver-preservation, transplantation, UW-solution, Euro-Collins

### INTRODUCTION

Until Belzer and co-workers[1] introduced a new solution for preservation of solid organs the preservation time for human liver was limited to 6–10 hours using a modified version of a solution introduced by Collins in 1969[2]. This modified version is called Euro-Collins solution and it was used in most countries in clinical kidney, liver and pancreas transplantation[3].

Preservation of rat liver after flushing with Euro-Collins and cold-storage in this medium for longer than 12 hours resulted in irreversible damage to sinusoidal cells and hepatocytes[4]. Many techniques have been investigated with the aim to prolong

Address correspondence to: Onno T. Terpstra, M.D., Dept. of Surgery, University Hospital Dijkzigt, Dr Molewaterplein 40, 3015 GD Rotterdam, The Netherlands.

the preservation period: prostacyclin infusion before ischemia[5,7], rapid flush methods[8], rapid cooling of the organ[9] and the use of different storage solutions[10,19].

By prolongation of the preservation period histocompatibility matching between the donor and the recipient becomes possible, the donor pool increases by enlarging the area from which donor livers can be obtained and liver transplantation becomes a semi-elective procedure instead of an emergency one.

Recently successful 24-hours-preservation of the liver using UW-solution, has been reported[11]. The aim of our study was to investigate the morphology and metabolic capacity of rat liver after 18 and 42 hours cold storage in UW-solution (UW) as compared with cold storage in Euro-Collins solution (EC).

### MATERIAL AND METHODS

Male Wistar rats, weighing 200–300 g, were randomly allocated to four groups: 18-hr storage UW solution ($n = 6$), 18-hr EC ($n = 6$), 42-hr UW ($n = 8$) and 42-hr EC ($n = 8$). Eight other rats were used as controls (no preservation). All rats were deprived of chow 48 hours prior to the isolation of the liver but had free access to water.

### *Surgical Procedures*

Anesthesia was induced by intraperitoneal injection of pentobarbital sodium (60 mg/kg body weight). The surgical and recirculating perfusion technique were as described earlier[20]. The perfusion medium was gassed with carbogen (95% $O_2$, 5% $CO_2$), 600 mL/min. After harvesting, the liver was transferred to the perfusion cabinet where it was perfused for 10 minutes with either one of the two solutions (UW or EC) at 4°C (Figure 1). After this preperfusion period the liver was stored at 4°C in 100 ml of either one of the preservation solutions for 18 or 24 hours. After this cold storage period the liver was placed back in the cabinet and the second perfusion period started with a 1 hour-period at 37°C with Krebs-Ringer solution. Livers of the control rats were used immediately after isolation without cold-storage. After adding 2nM L-triiodothyronine and $^{125}$Iodine-triiodothyronine ($7 \times 10^6$ counts) to the perfusion medium, the perfusion was continued for another hour (experimental period).

### *Liver Tests*

Function of the liver was evaluated by several parameters: during the first hour of perfusion ASAT-activity was determined 10 and 55 minutes after starting; the perfusion pressure as determined by the height of the perfusion column (to obtain a medium flow of 40 mL/min through the liver); the amount of bile secretion, which was measured every 10 min during the experimental period and histological changes in a wedge biopsy of liver tissue taken at the end of the experiment. Histological evaluation was performed by an observer who had no knowledge of the kind of storage solution that was used. Histological examination concerned the architecture of the whole liver tissue specimen as well as degenerative changes of the hepatocytes, the cohesion between the hepatocytes, changes in cytoplasm and nucleus, the appearance of the portal fields (bile ducts, edema, inflammatory infiltration, bleeding, etc.) and the amount of glycogen stored in the cells.

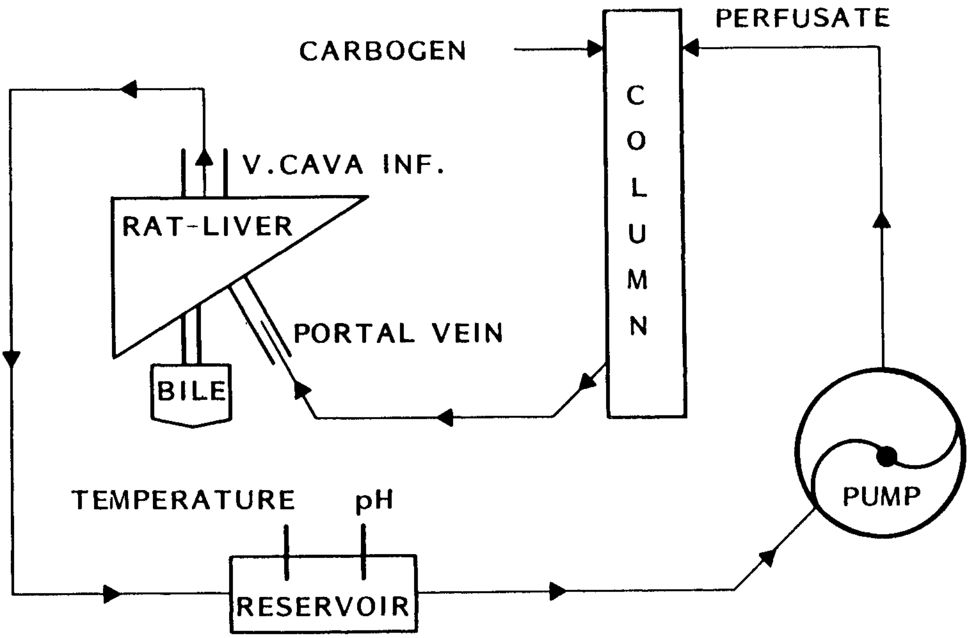

**Figure 1** Schematic schedule of the isolated rat liver perfusion model.

### *Triiodothyronine-kinetics*

To evaluate the metabolic capacity of the hepatocytes, triiodothyronine-kinetics were studied. Two components were investigated: transport of triiodothyronine over the hepatocyte cell membrane and the deiodination and conjugation of triiodothyronine. These two phases of thyroid hormone metabolism are both energy-consuming processes and are sensitive parameters of liver function[21,22].

Aliquots of the perfusion medium were taken 0.5 min after the start of the experimental period and at one minute intervals until 10 min after start of the experimental period, then at 5–10 min intervals until one hour after the start. Perfusate and bile samples were stored at –20°C until further analysis. All samples were analysed by chromatography on small Sephadex LH-20 columns equilibrated with 0.1 M HCl. Iodide was eluted with HCl; triiodothyronine-glucuronide, triiodothyronine-sulfate and triiodothyronine with sodium-acetate, $H_2O$ and NaOH/ethanol (50/50 vol/vol) respectively.Fractions of 1 ml were collected and counted for radioactivity. For analysis of triiodothyronine-kinetics the tracer triiodothyronine disappearance curve from medium was fitted into a two pool model developed by Doctor *et al.*[23]. This model enables discrimination between triiodothyronine-transport and triiodothyronine-metabolism in general in the rat liver.

Statistical evaluation of the experiments was performed by the Mann-Whitney U-test. Results are presented as mean ± standard error of the mean.

### RESULTS

### *Macroscopic Appearance*

At the end of the experiments there were slight macroscopic differences between the four cold-storage groups of livers. All livers stored in UW were less pale and showed fewer white ischemic spots than livers stored in Euro-Collins. The differences between the groups were more pronounced after 42-hr than after 18-hr cold-storage.

### *ASAT-activity*

In the UW-stored group (18 and 42-hr) ASAT-levels were significantly lower at 10 and 55 min (in the first hour of perfusion) as compared with EC-stored (18 and 42-hr) livers. In the control group ASAT-levels at 10 min were lower than in the 42-hr EC group, whereas at 55 min ASAT-levels in the control group were lower than in the UW (42-hr) and in both EC groups (Table 1.).

**Table 1** ASAT-activity in the perfusate at 10 min and 55 min (in the first hour of perfusion). Perfusion pressure as determined by the height of the perfusion column to obtain a perfusate flow of 40 mL/min. Triiodothyronine-metabolism ($T_3$-metab.) and triiodothyronine-transport ($T_3$-trans.) as calculated by the two pool model[6]. Values presented as mean ±SEM.

| | *ASAT (U/L) 10 min* | *ASAT (U/L) 55 min* | *Perfusion pressure (cm)* | *$T_3$-metab. (pmol/pmol)* | *$T_3$-transp. (1/min)* |
|---|---|---|---|---|---|
| Controls (n = 8) | 5 ± 0.9 | 19 ± 3.6 | 8.3 ± 0.3 | 0.139 ± 0.02 | 0.38 ± 0.08 |
| UW 18 hr (n = 6) | 2.6 ± 1.2[ab] | 13.8 ± 4.2[bf] | 9.1 ± 0.5 | 0.118 ± 0.02 | 0.24 ± 0.02[l] |
| EC 18 hr (n = 6) | 8 ± 1.7[c] | 44.3 ± 5.8[gh] | 11.8 ± 0.2[k] | 0.111 ± 0.02 | 0.27 ± 0.03[k] |
| UW 42 hr (n = 8) | 7.8 ± 0.9[d] | 46.9 ± 5.9[ij] | 8.8 ± 0.6[j] | 0.117 ± 0.01[j] | 0.24 ± 0.04[l] |
| EC 42 hr (n = 8) | 21.9 ± 5.0[e] | 84.2 ± 14.5[e] | 10.8 ± 0.5[e] | 0.097 ± 0.01[e] | 0.25 ± 0.03[e] |

a = UW 18 vs EC 18: $P < 0.05$
b = UW 18 vs UW 42: $P < 0.01$
c = EC 18 vs EC 42: $P < 0.01$
d = UW 42 vs EC 42: $P < 0.01$
e = EC 42 vs Controls: $P < 0.01$
f = UW 18 vs EC 18: $P < 0.01$
g = EC 18 vs Controls: $P < 0.01$
h = EC 18 vs EC 42: $P < 0.05$
i = UW 42 vs Controls: $P < 0.01$
j = UW 42 vs EC 42: $P < 0.05$
k = EC 18 vs Controls: $P < 0.05$
l = UW 18 vs Controls: $P < 0.05$

### *Triiodothyronine-kinetics*

Triiodothyronine-transport was similar in the EC and UW-stored livers. The livers in all cold-storage groups had significantly lower values than those in the control group. Triiodothyronine-metabolism in the control group and in the UW (42-hr) group were significantly better than in the EC (42-hr) stored livers (Table 1.).

*Perfusion Pressure*

The perfusion pressure was similar in the UW (18 and 42-hr) and the control group. The perfusion pressure in the EC (18 and 42-hr) livers was higher than in the control group. After 42-hr cold-storage in EC the perfusion pressure was higher than after 42-hr cold storage in UW. After 18-hr cold-storage the perfusion did not differ (Table 1.).

*Bile Secretion*

Total bile production during the experimental period was significantly higher in UW (42-hr) stored livers than in the EC (42-hr) stored group, whereas after 18 hr cold storage the bile production did not differ. Livers in all cold-storage groups (except UW 18-hr) secreted less bile as compared with control livers (Table 1.)

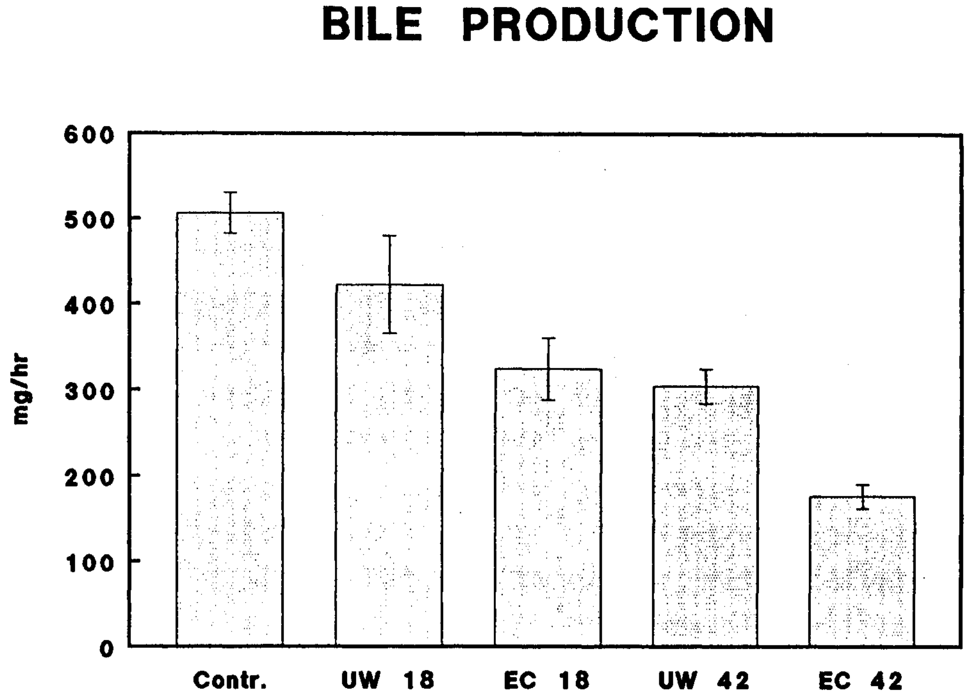

**Figure 2** Total bile production in the experimental period (mean ± SEM). Contr. = control group (no cold storage); UW 18, UW 42 = 18-hr or 42-hr cold-storage in UW-solution; EC 18, EC 42 = 18-hr or 42-hr cold-storage in Euro-Collins solution. Controls vs. EC 18, UW 42 and EC 42: $P<0.01$, EC 18 vs. EC 42: $P<0.01$, UW 42 vs. EC 42: $P<0.01$.

*Histology*

In general UW-stored livers had less histological changes than EC-stored livers, in the 18-hr as well as in the 42-hr cold storage groups. In 2/6 EC (18-hr), in 0/6 UW (18-hr), in 5/8 EC (42-hr) and in 1/8 UW (42-hr)-livers more than 10% of the hepatocytes were necrotic. None of the livers in the control group showed more than 10% necrosis. Edema in the portal triads was less evident in UW-stored (18 and 42-hr) livers. Degenerative changes of the liver-parenchyma (such as diminished cohesion with rounded hepatocytes, pale vacuolated cytoplasm with eosinophilic changes, pycnosis of nuclei and nuclear remnants in Kupffer-cells and in sinusoidal vessels) were less pronounced in UW-stored (18 and 42-hr) livers as compared with EC-stored (18 and 42-hr) livers. Livers in the control group had a nearly normal histological appearance with only a few hepatocytes showing degenerative changes. There were no evident differences in glycogen content in the hepatocytes, which was diminished in the control, the UW (18-hr) and the EC (18-hr) group. In the UW (42-hr) and the EC (42-hr) group the amount of glycogen was severely diminished. In two thirds of the livers in both 42-hr cold-storage groups bacterial colonies were found, whereas in the control, the UW (18-hr) and the EC (18-hr) group no bacterial colonies were observed.

### DISCUSSION

The aim of our study was to evaluate two cold storage solutions, the Euro-Collins-solution and the newer UW-solution in the isolated rat liver perfusion model. The isolated liver perfusion model is a good alternative to studies in which the liver is grafted for assessment of preservation solutions[11]. In transplantation experiments immunological and technical problems may obscure the overall results. The isolated rat liver model is a simple, rapid and inexpensive way to assess liver function without interference from other organ systems in the body, while the integrity of the liver is not affected.

ASAT-levels in the perfusate differed significantly between the 18-hr cold-storage groups, and between the 42-hr cold-storage groups, but showed considerable intra-group variability. The latter may be due to continuous perfusion with the same perfusate which can cause accumulation to the effects of these values. Other groups have also found great variation in ASAT-levels and stated that this parameter in the perfusate was not related to later liver function in humans[24] or in rabbits[11].

More sophisticated methods to monitor liver function are available nowadays, one of these is assessment of thyroid hormone metabolism. Thyroid hormone transport and intracellular-metabolism are energy consuming processes and therefore dependent on the availability of energy stores[21,22,25,26]. It has been shown that in conditions of reduced ATP-availability (in primary rathepatocyte cell cultures) both triiodothyronine transport and metabolism are decreased[23]. Our results showed a similarly decreased transport of triiodothyronine after cold storage in both UW and EC groups, whereas triiodothyronine-metabolism is more diminished after 42-hr cold-storage in either EC or UW than after 18-hr cold-storage in these solutions, indicating that triiodothyronine-metabolism is a valuable monitor of the metabolising capacity of the liver.

The perfusion pressure is a reflection of the amount of tissue edema. The perfusion pressure in UW (42-hr) stored livers was significantly lower than that of livers stored in EC (42-hr), whereas the perfusion pressure in the UW (18-hr) and in the EC (18-hr) groups did not differ. This finding is consistent with Belzer's concept of adding raffinose and hydroxyethyl starch to the cold-storage solution to prevent expansion of the extracellular space[28].

As other groups have also asserted we confirm that bile production is a simple and reliable monitor of liver function[11,24,29–31]. When we compare bile production with perfusion pressure and triiodothyronine-kinetics, the differences between the groups are very consistent.

Because of the high metabolic rate, hepatic cells are most sensitive to the deleterious effects of anoxia[32]. Therefore light microscopic examination of liver tissue (biopsies) has proved to be a good monitor of ischaemic damage to the cells[4,14,19,33,34]. Others have found light microscopic investigation to be of limited value[35]. In our study we found a good correlation between the histological appearance and the function of the hepatocytes. The amount of glycogen in the hepatocytes was not a reliable monitor of liver condition after 42-hr cold-storage. The decreased amount of glycogen could also be due to the 48-hr fasting period preceding the cold-storage period, because hepatocytes in the control group and in the 18-hr cold storage groups also contained less glycogen.

Summarising these results we conclude that in the isolated rat liver perfusion model cold-storage with UW-solution gave better metabolic performance and morphological results than with Euro-Collins solution. The differences between UW-solution and Euro-Collins solution are more pronounced after 42-hr than after 18-hr cold storage.

### *References*

1. Wahlberg, J.A., Love, R., Landegaard, I., Southard, J.H. and Belzer, F.O. (1987) 72-hour preservation of the canine pancreas. *Transplantation*, **43**, 5–8
2. Collins, G.M., Bravo-Shugarman, M. and Terasaki, P.I. (1969) Kidney preservation for transportation. Initial perfusion and 30-hours ice storage. *Lancet*, **2**, 1219–1222
3. Dreikorn, K., Horsch, R. and Rohl, L. (1980) 48 to 96 hour preservation of canine kidneys by initial perfusion and hypothermic storage using the Euro-Collins solution. *Eur. Urol.*, **6**, 221–224
4. Myagkaya, G.L., van Veen, H. and James, J. (1987) Ultrastructural changes in the rat liver during Euro-Collins storage, compared with hypothermic *in vitro* ischemia. *Virchows Arch B*, **53**, 176–182
5. Monden, M. and Fortner, J.G. (1982) 24 and 48-hour canine liver preservation by simple hypothermia with prostacyclin. *Ann. Surg.*, **196**, 38–42
6. Mora, N.P., Cienfuegos, J.A., Pereira, F. *et al.* (1988) Value of $PGI_2$ plus verapamil for obtaining 24-hour preserved liver allografts. *Transplant Proc.*, **20**, 980–982
7. Todo, S., Yokoi, H., Podesta, L. *et al.* (1988) Amelioration of normothermic canine liver ischemia with $PGI_2$. *Transplant Proc.*, **20**, 965–968
8. Miller, C., Mazzaferro, V., Makowka, L. *et al.* (1988) Rapid flush technique for donor hepatectomy: safety and efficacy of an improved method of liver recovery for transplantation. *Transplant Proc.*, **20**, 948–950
9. Otto, G., Wolff, H., Uerlings, I. and Gekert, K. (1986) Preservation damage in liver transplantation, influence of rapid cooling. *Transplantation*, **42**, 122–124
10. Bergren, C., Toledo-Pereyra, L.H., Bandlien, K.O. and MacKenzie, G.H. (1986) Comparison of preservation solutions and methods for liver transplantation. *Transplant Proc.*, **18**, 592–593
11. Jamieson, N.V., Sundberg, R., Lindell, S., Southard, J.H. and Belzer, F.O. (1988) A comparison of cold storage solutions for hepatic preservation using the isolated perfused rabbit liver. *Cryobiology*, **25**, 300–310
12. Lie, T.S. and Ukikusa, M. (1984) Significance of alkaline preservation solutions in liver transplantation. *Transplant Proc.*, **16**, 134–137
13. Otto, G., Wovlff, H. and David, H. (1984) Preservation damage in liver transplantation: electron microscopic findings. *Transplant Proc.*, **16**, 1247–1248

14. Otto, G., Wolff, H. and Häcker, R. (1984) Adenine nucleotides and glycolysis during liver preservation by simple hypothermic storage. *Eur. Surg. Res.*, **16**, 84–88
15. Otto, G., Wolff, H. and David, H. (1986) Graft damage in liver transplantation. *Transplant Proc.*, **18**, 1201–1202
16. Tamaki, T., Kamada, N. and Pegg, D.E. (1986) Hypothermic preservation of the rat liver assessed by orthotopic transplantation, a comparison of flush solutions. *Transplantation*, **41**, 396–397
17. Tamaki, T., Kamada, N. and Pegg, D.E. (1987) Hypothermic preservation of the rat liver assessed by orthotopic transplantation. II Evaluation of citrate solutions. *Transplantation*, **43**, 357–361
18. Toledo-Pereyra, L.H., Castellanos, J. and Chapman, M. (1987) Failure to preserve liver allografts for 24 hours: experimental and theoretical considerations. *Transplant Proc.*, **19**, 68–70
19. Vine, W., Gordon, E., Alger, J. and Flye, M.W. (1986) Hepatic preservation assessed by magnetic resonance spectroscopy. *Transplant Proc.*, **18**, 577–581
20. Meijer, D.K.F., Keulemans, K. and Mulder, G.J. (1981) Isolated perfused rat liver technique. *Methods in Enzymology*, **77**, 81–94
21. Dills, R.L. and Klaassen, C.D. (1986) The effect of inhibitors of mitochondrial energy production on hepatic glutathione, UDP-glucuronic acid and adenosine 3'phosphate-5'phosphosulfate concentration. *Drug Metab. and Disposition*, **14**, 190–196
22. Krenning, E.P., Docter, R., Bernard, H.V., Visser, T.J. and Hennemann, G. (1981) Characteristics of active transport of thyroid hormone into rat hepatocytes. *Biochem. Biophys. Acta*, **676**, 314–320
23. Docter, R., Jong, M. de, Hoek, H.J. van der, Krenning, E.P. and Hennemann, G. (1990) Development and use of a mathematical two pool model of distribution and metabolism of 3,3',5-triiodothyronine in a recirculating rat liver perfusion system: albumin does not play a role in cellular transport. *Endocrinology*, **126**, 1–9
24. Kamiike, W., Burdelski, M., Steinhoff, G., Ringe, B., Lauchart, W. and Pichlmayr, R. (1988) Adenine nucleotide metabolism and its relation to organ viability in human liver transplantation. *Transplantation*, **45**, 138–143
25. Krenning, E.P., Docter, R., Bernard, H.F., Visser, T.J. and Hennemann, G. (1978) Active transport of triiodothyronine ($T_3$) into isolated rat liver cells. *FEBS Lett.*, **91**, 113–116
26. Krenning, E.P., Docter, R., Bernard, H.F., Visser, T.J. and Hennemann, G. (1982) Decreased transport of thyroxine ($T_4$), 3,3',5-triiodothyronine ($T_3$) and 3,3',5'-triiodothyronine ($rT_3$) into rat hepatocytes in primary culture due to a decrease of cellular ATP content and various drugs. *FEBS Lett.*, **140**, 229–233
27. Heyden, J.T.M. van der, Docter, R., Toor, H. van, Wilson, J.H.P. Hennemann, G. and Krenning, E.P. (1986) Effects of caloric deprivation on thyroid hormone tissue uptake and generation of low-$T_3$ syndrome. *Am. J. Physiol.*, **251**, E156–E163
28. Belzer, F.O. and Southard, J.H. (1988) Principles of solid organ preservation by cold storage. *Transplantation*, **45**, 673–676
29. Kamada,N., Calne, R.Y., Wight, D.G.D. and Lines, J.G. (1980) Orthotopic rat liver transpslantation after long-term preservation by continuous perfusion with fluorocarbon emulsion. *Transplantation*, **30**, 43–48
30. Kamiike, W., Nakahara, M. Nakao, K. *et al.* (1985) Correlation between cellular ATP level and bile excretion in the rat liver. *Transplantation*, **39**, 50–55
31. Lee, D. and Holland, R.K. (1979) Improved performance of the isolated rat liver when perfused with purified bovine serum albumin. *Transplantation*, **27**, 384–388
32. Marubayashi, S., Takenaka, M., Dohi, K., Ezaki, H. and Kawasaki, T. (1980) Adenine nucleotide metabolism during hepatic ischemia and subsequent blood reflow periods and its relation to organ viability. *Transplantation*, **30**, 294–296
33. Caldwell-Kenkel, J.C., Thurman, R.G. and Lemasters, J.J. (1987) Reperfusion injury to endothelial cells following cold ischemic storage of rat liver in Euro-Collins solution. *Hepatology*, **7**, 1048
34. Myagkaya, G., van Veen, H. and James, J. (1984) Ultrastructural changes in rat liver sinusoids during prolonged normothermic and hypothermic ischemia *in vitro*. *Virchow Arch (Cell Pathol)*, **47**, 361–373
35. Tamaki, T., Kamada, N., Wight, D.G. and Pegg, D.E. (1987) Successful 48-hour preservation of the rat liver by continuous hypothermic perfusion with haemacel-isotonic citrate solution. *Transplantation*, **43**, 468–471

*(Accepted by S. Bengmark 23 April 1991)*

